# Toward Fully Photoresponsive Amphiphilic Polymers via Azopyrazole-Functionalized Polyacrylamides

**DOI:** 10.1021/acs.macromol.5c01176

**Published:** 2025-10-08

**Authors:** René Steinbrecher, Florian Lehmann, Ruslan Nedielkov, Tillmann Klamroth, Heiko Möller, Andreas Taubert, Dariush Hinderberger, Peter Müller-Buschbaum, Christine M. Papadakis, André Laschewsky

**Affiliations:** a Institute of Chemistry, 26583 University of Potsdam, Potsdam-Golm 14476, Germany; b Institute of Chemistry, 9176 Martin Luther University Halle-Wittenberg, Halle (Saale) 06099, Germany; c Department of Physics, TUM School of Natural Sciences, Technical University of Munich, Garching 85748, Germany; d Fraunhofer Institute for Applied Polymer Research IAP, Potsdam-Golm 14476, Germany

## Abstract

Arylazopyrazoles (AAPs) are a rapidly emerging class of photoswitches, which stand out due to their distinct absorption maxima of the *E-* and Z-isomers, the highly *Z*- or *E-*isomer-rich photo stationary states upon irradiation by UV- or visible light, respectively, and the long lifetime of the metastable *Z*-state. Here, we present water-soluble acrylamide copolymers functionalized by an AAP dye. This renders them both thermo- and photoresponsive and enables the reversible modulation of their LCST-type phase transition temperature (PTT) in aqueous solution by the *E-* to Z-isomerization. By alternating irradiation with UV and green light, the PTT is modulated effectively and can be shifted for optimized AAP contents by up to 27 °C, i.e., much higher than by common azobenzene photoswitches. The AAP photoswitch and the transition process of the dye-modified polymer are investigated using density functional theory calculations, turbidity measurements, and temperature-dependent NMR, EPR, and UV–vis spectroscopies.

## Graphical abstract

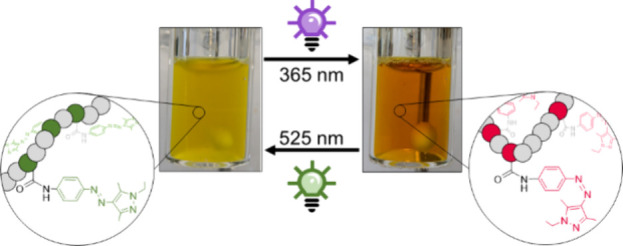

## Introduction

Polymers that undergo large and reversible changes in their properties following a relatively small trigger event are referred to as ″stimuli-responsive polymers″. Of particular interest is the solubility-insolubility phase transition of such polymers in aqueous solution and the associated changes in their conformation.^1^ Common triggers for such systems are, e.g., small changes of the pH-value^2^ or salt concentration,^3^ or the temperature.^4^ Such “smart materials” have been explored for various application fields, for instance, sensors, targeted drug delivery, rheology modifiers, artificial muscles, or actuators.^5–8^

Thermoresponsive polymers typically exploit a temperature-modulated miscibility gap. They can be classified into polymers featuring an upper critical solution temperature (UCST) or a lower critical solution temperature (LCST), with the majority of systems reported showing LCST behavior in aqueous solution for noncharged polymers.^9^ In fact, many nonionic polymers, e.g., poly­(vinyl alcohol), poly­(ethylene oxide), and many of their derivatives,^10^ as well as numerous polyoxazolines^11^ or poly­(meth)­acrylamides,^12^ show LCST behavior, which is typically characterized by the cloud point temperature (*T*_CP_) at a specific concentration.^8^ Compared to changes in pH or salt concentration, a change of temperature has the advantage of being a noninvasive trigger, i.e., no additional compounds (e.g., acids, bases, or salts) need to be added to induce switching, nor do such substances need to be laboriously separated from the system (e.g., via dialysis) to revert to their solution state. Therefore, noninvasive triggers allow a priori for convenient and facile back-and-forth switching.

Although much less explored than temperature changes, light irradiation is another noninvasive stimulus that offers additional benefits due to its high temporal and spatial resolution. Photoresponsive polymers in aqueous media can be realized by the incorporation of photoreactive groups that change their polarity or hydrophilicity upon undergoing a photoisomerization process. The most common examples are spiropyrans,^13,14^ donor–acceptor Stenhouse adducts (DASAs),^15^ cinnamates^16,17^ or above all, azo dyes, most commonly in the form of azobenzenes.^18–22^ Usually, such polymers combine photo- with thermoresponsive behavior.^18^ Instead of crossing the soluble–insoluble phase boundary line by modulating the temperature, the phase transition temperature (PTT) is modulated via the photoreaction of the photoactive group. The resulting changes of their dipole moment, and therefore of the polarity,^19^ shift the position of the miscibility gap in the phase diagram, thus ensuring a switch of the water-solubility within a specific temperature window under isothermal conditions. Moreover, within this temperature window, both temperature and light can be used orthogonally as triggers to induce the LCST-type phase transition.

Azobenzenes, alike all common azo dyes, can be isomerized with UV-light of a specific wavelength from the a priori thermodynamically favored *E-*isomer (*trans*) to the metastable Z-isomer (*cis*).^20^ In contrast to the *E-*isomer, the *Z*-isomer of azobenzene possesses a significant dipole moment,^19^ which reduces its hydrophobicity, and spontaneously relaxes to the *E-*state over time. As *E-* and *Z*-isomers have distinct absorbance spectra, the back reaction from the *Z*- to the *E-*state can alternatively be induced by irradiation with light of a different specific wavelength that is typically located in the visible range. Importantly, the photoisomerization of azobenzene-based systems is generally not subject to side reactions and, therefore, shows no fatigue of the photoswitch.^21^ For this reason, azobenzene has been widely used in stimuli-responsive systems, e.g., *T*_CP_ modulation of aqueous copolymers^22–25^ or the photomodulated swelling of hydrogels.^26–28^

Despite its merits, the use of azobenzene photoswitches in water-soluble polymers is hampered by several difficulties. On the one hand, the only moderate change of polarity/hydrophilicity upon *E-Z*-isomerization requires incorporating a high dye content to achieve effective photoswitching. On the other hand, the intrinsic hydrophobic character of the chromophore limits the maximum content of photoreactive groups that can be practically incorporated to achieve water-solubility at all. Moreover, the absorption bands of the *E-* and *Z*-isomers overlap considerably,^21,29^ resulting in a mixture of isomers in the photostationary state (PSS) that may reduce the achievable polarity contrast even further. As a result, *T*_CP_ modulation by azobenzene photoswitches has been mostly limited to a few degrees.^25,30–37^ Only in exceptional cases modulations by 10–15 °C have been reported.^29,38–40^ Also, when bistable switching scenarios are aspired, the metastability of the *Z*-state may pose problems, as its half-life is often quite short (*t*_1/2_ < 1 h).^41^ Noteworthy, the *E-* to *Z*-isomerization of azo dyes does not always result in an increase of the PTT, but occasionally, a counterintuitive decrease is observed after irradiation with UV-light, the reasons not being fully clear yet.^24,33,40,42,43^ *A priori*, it seems not critical for a specific application whether the photoinduced shift is due to the *E-*to-*Z* or to the *Z*-to-*E-*isomerization of the azo dye, yet these findings exemplify that the triggering effect of the azo chromophore is not yet fully understood.

The limitations of azobenzene-based systems discussed above have led to the development of new types of azo dyes with better resolved or more strongly red-shifted absorption maxima for both isomers, and with extended lifetimes of the metastable *Z*-isomer.^20,21,44^ For instance, the class of aryl azopyrazole (AAP) photoswitches introduced by Fuchter and co-workers can overcome many of the above-mentioned limitations, exhibiting half-lives of the *Z*-isomer ranging from 1 to 1000 d and distinct absorption maxima for both the *E*- and *Z-*isomers.^45^ This allows for almost quantitative and effective switching between the two states using UV and green light.^45^ These efficient photoswitches have since been explored in various scenarios, including the surface functionalization of glass,^46^ metal oxides^47,48^ or gold^49^ as well as the synthesis of photocontrolled gelators,^50^ shape-memory materials,^51^ host–guest systems,^52,53^ and enzyme inhibitors.^54^ Most recently, AAP-functionalized amphiphilic block copolymers were studied with respect to their photomodulated self-assembly in water, implementing a macro surfactant-like structure by copolymerizing a poly­(ethylene glycol) methacrylate block with an AAP-functionalized polymethacrylate block via RAFT polymerization.^55,56^ The ability of the block copolymers to change the form of their self-assembled morphology from spheres to rods or stretched spheres by light irradiation was reported.^55,56^ In parallel, the modulation of *T*_CP_ of LCST-based thermoresponsive polymers using AAP switches was recently introduced.^57^

In our initial communication, we reported on the general properties of the AAP-bearing monomer arylazopyrazole ethyl acrylamide (AAPEAm), as well as on the influence of the AAPEAm monomer content in a series of copolymers with *N,N*-dimethylacrylamide (DMAm).^57^ Most noteworthy, we observed exceedingly high shifts of *T*_CP_ after *E-Z* isomerization upon UV-irradiation, reaching almost 25 °C. The magnitude of the *T*_CP_ shift, Δ*T*_CP_, initially increased with the amount of incorporated chromophore, but eventually passed through a maximum to finally decline for higher dye contents. The maximum switch was obtained for a dye content of around 7 mol %. In this follow-up study, we present continuative investigations of the photoswitch monomer and the polymer at optimum dye content using complementary techniques such as temperature-resolved turbidimetry, electron paramagnetic resonance (EPR), and nuclear magnetic resonance (NMR) spectroscopy, to gain further insight into its switching behavior from the macroscopic down to the molecular level. In particular, we establish that the arylazopyrazole-functionalized polymer exhibits advantageous spectroscopic properties, e.g., in regard to the resolution of the π → π* transition maxima of both isomers, similar to the recently reported ones for low molar mass members of this new azo dye family.^45^ This enabled us to establish the fully bidirectional photoswitching between both photoisomeric states with nearly 100% selectivity, by alternate irradiation with UV- and visible light of specific wavelengths.

## Materials and Methods

### Chemicals

Deuterated sodium trimethylsilylpropanesulfonate (DSS-D_6_, 98 atom% D) was purchased from Merck. 5-DOXYL stearic acid, ammonium salt (5-DSA, or 2-(3-carboxypropyl)-2-tridecyl-4,4-dimethyl-3-oxazolidinyloxyl, > 99% purity) was purchased from Avanti Polar Lipids (Alabaster, AL, USA). Deuterated water and methanol were purchased from VWR Chemicals (99.8% D) and used as received. Methanol used for EPR-experiments was HPLC-grade (>99.9% purity, Sigma-Aldrich, Taufkirchen, Germany). The syntheses of the monomer (E)-*N*-(4-((1-ethyl-3,5-dimethyl-1H-pyrazole-4-yl)­diazenyl)­phenyl)­acrylamide (AAPEAm) and its copolymers with *N,N*-dimethylacrylamide (DMAm) were described previously.^57^

### Light Sources Used for Sample Irradiations for Turbidimetry, UV–vis, and NMR Spectroscopy Studies

UV-light irradiation was performed with a 365 nm UV LED flashlight, Alonefire SV47 12 W (Shenzhen Shiwang Technology Co. Ltd., Shenzhen, China). The samples were irradiated at an irradiance of 200 mW·cm^–2^ as measured by an optical power meter PM100D with a sensor S170C (Thorlabs, Newton, USA). Blue and green light irradiations were performed with a home-assembled setup using a 3 W high-power LED as irradiation source from LEDs-and-more (Berlin, Germany).

### DFT Calculations

All calculations are performed with the double-hybrid-meta-GGA density functional PWPB95-D3 and the def2-TZVP basis set as provided by orca 6.0.1.^58^ We chose PWPB95-D3 because it was found to be the top functional for dipole moment predictions.^59^ All structures are fully optimized and confirmed as minima on the potential energy surface by frequency calculations.

### UV–vis and Turbidimetry

Measurements were performed on a dual-beam spectrometer, Model Carry500 (Agilent). Samples (concentration 10 g·L^–1^ in water or deuterated water) for turbidimetry were prepared at least 1 day before the measurement, and were annealed at room temperature in the dark. The samples were heated at a rate of 0.5 K min^–1^, and the transmission at a wavelength of 600 nm was recorded every 0.25 K. The cloud point (*T*_CP_) was defined as the onset of rapid decay of the transmission.

### Liquid-State Nuclear Magnetic Resonance (NMR) Spectroscopy for Isomer Ratio Determination

NMR spectra for the quantitative determination of the *E-* to *Z-* state ratio were recorded on a Bruker AVANCE NEO 400 MHz spectrometer equipped with a BBO 400S1 BBF-H-D-05 Z SP probe at 25 °C in deuterated methanol as a solvent (concentration = 17 g·L^–1^). ^1^H NMR spectra (16 scans with 20.48 ppm spectral width, 4 s acquisition time, and 1 s recycling delay) were acquired and evaluated using TopSpin software, version 4.3.0. The ratio of both isomers was determined by integrating the singlet signals of the methyl groups of the pyrazole moiety at 2.6 and 2.5 ppm (*E-*isomer), and at 2.1 and 1.6 ppm (*Z*-isomer) (examples are shown in the Supporting Information, Figures S1 and S2). The samples were irradiated in an NMR glass tube for 15 min, independent of the wavelength used (λ = 365, 450, 480, or 525 nm).

### Temperature-Dependent Electron Paramagnetic Resonance (EPR) Spectroscopy under Light Irradiation

A monochromatic irradiation setup within the MS5000 benchtop EPR spectrometer (Magnettech GmbH, Berlin/Freiberg Instruments, Freiberg, Germany – now part of Bruker Biospin, Ettlingen, Germany) was used. Micropipettes (BLAUBRAND intraMARK, Wertheim, Germany) were filled with around 10 μL of sample solution (concentration = 10 g·L^–1^ in water) polymer and 100 μM 5-DSA. Monomer AAPEAm was dissolved in methanol due to its low solubility in water. The capillary tube sealant (CRITOSEAL Leica) was used to close the sample tubes. The light-induced isomerization experiments were performed at 25 °C (±0.2 °C). For recording a temperature series, the temperature inside the spectrometer was set to 15 °C (±0.2 °C) and gradually increased to 75 °C in steps of 5 °C. Before starting each measurement, the sample was equilibrated at each temperature for 2 min. A magnetic field sweep of 12 mT centered around 338 mT with a scan time of 60 s, a modulation amplitude of 0.1 mT (100 kHz) and a microwave power of 4.8 mW was used to obtain EPR spectra. Each spectrum is an accumulation of 10 scans. EPR spectral simulations were performed using the EasySpin software package^60^ and the “chili” function, which describes slow, nonisotropic tumbling of nitroxide radicals based on the Schneider-Freed theory.^61^

### Temperature-Resolved Liquid-State NMR Spectroscopy

All spectra were acquired on a Bruker AVANCE NEO 500 MHz spectrometer equipped with a nitrogen cryogenically cooled TCI 500S2 H&F–C/N-D-05 Z XT probe and processed using TopSpin 4.3.0. The copolymer p­(DMAm-*stat*-AAPEAm) was dissolved in D_2_O (concentration 10 g·L^–1^), and perdeuterated sodium trimethylsilylpropanesulfonate (DSS-D_6_) was added to the final concentration of 10 mM. DSS-D_6_ was used as an internal reference for the ^1^H chemical shift (0 ppm), and the integral of the signal was used as a reference to quantify the decay of the polymer signals between 3.5 and 0.5 ppm and between 8.2 and 6.8 ppm. A series of ^1^H NMR spectra (64 scans with 20 ppm spectral width, 3.3 s acquisition time, and 1 s recycling delay) was recorded as a function of the temperature, after annealing in the dark as well as after irradiation with 365 nm light. Starting at 20 °C, the temperature was increased in 5 °C steps until 80 °C was reached. Before each measurement, the new temperature was held for 20 min to thermally equilibrate the sample. The sample was then cooled to 20 °C and irradiated at 365 nm for 15 min, and the above procedure was repeated with one additional interruption to irradiate the sample, after half of the planned ^1^H NMR spectra were recorded.

## Results & Discussion

### Properties of the Photoswitch

The chemical structure of the studied statistical copolymer p­(DMAm-*stat*-AAPEAm) is shown in Scheme 1. It is mainly composed of the hydrophilic monomer DMAm, containing about 7 mol % of the AAP-functionalized acrylamide AAPEAm (for the chemical structure of the monomer, see. Figure 2).

**Scheme 1:**
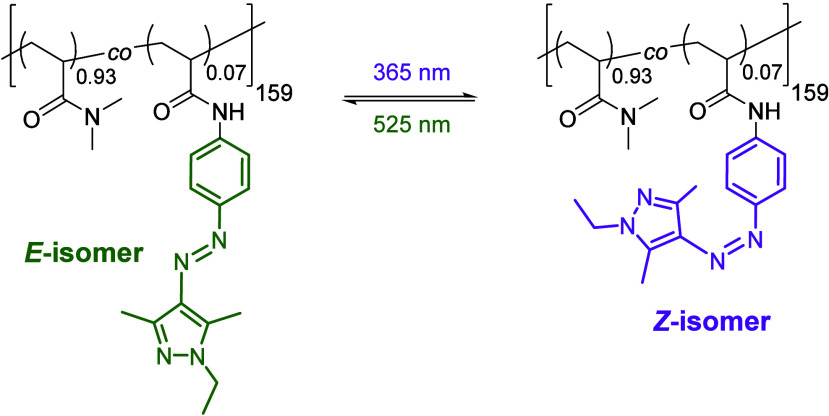
Chemical formulas of p­(DMAm-*stat*-AAPEAm) in both isomeric forms^a^

a The AAP chromophore can switch between the *E-* (in green) and *Z-*isomer (in purple) upon irradiation by near-UV and green light, respectively.

Note that the monomer AAPEAm is hydrophobic and insoluble in water, while the hydrophilic DMAm repeat units ensure the water-solubility of the copolymer. Still, the solubilizing capacity of the nonionic building block DMAm is limited. Therefore, copolymers with an AAPEAm content higher than 12 mol % are insoluble in water at any temperature at ambient pressure.^57^ As mentioned above, the standard explanation for the *T*_CP_ shift upon irradiation is due to the polarity increase of the *Z*-isomer compared to the *E-*isomer. Still, this argument is primarily based on considerations for classical azobenzenes and does not necessarily hold for the nonsymmetric azopyrazoles that are used in this study. For this reason, we calculated the dipole moment for a simplified aryl azopyrazole moiety in the *E-* and *Z-* state and compared it with the unsubstituted classical azobenzene (cf. Figure S3). The results for the azobenzene calculations are in agreement with the literature-reported change from 0 D for the *E*-isomer to around 3.3 D for the *Z*-isomer,^19^ showing a pronounced difference between both isomers that underpins the common explanation. The calculated dipole moments of the phenyl azopyrazole, on the other hand, show considerably smaller differences between both isomeric states, which are markedly affected by the orientation of the pyrazole group relative to the diazo bond and the aryl ring, as shown in Figure 1.

**Figure 1:**
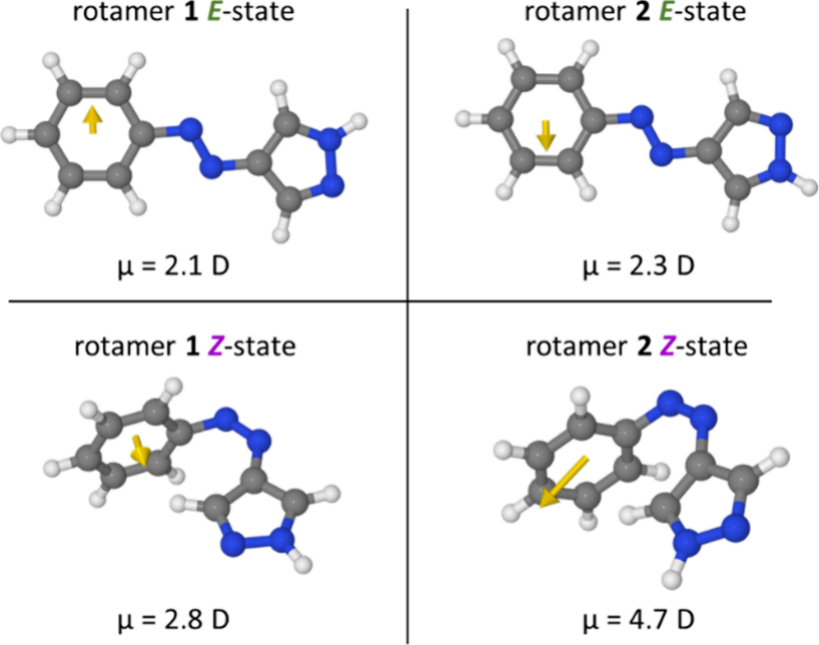
Calculated dipole moments for 2 rotamers of the 4-phenylazopyrazole chromophore with different orientations of the pyrazole with respect to the phenyl moiety, as indicated by the yellow arrows. Carbon is depicted in gray, nitrogen in blue, and hydrogen in white. Rotamer 1 in both isomeric states is shown on the left, and rotamer 2 in both isomeric states is shown on the right side.

As can be seen in Figure 1, the dipole moment changes for both rotamers are significantly smaller than the difference for classical azobenzene. While rotamer 1 (left side) shows a dipole moment increase of 0.7 D upon *E*-to-*Z* isomerization, rotamer 2 (right side) achieves an increase of 2.4 D. Thus, for rotamer 1, the absolute dipole moment change is even less than one-fourth of that of azobenzene. Moreover, the relative difference between both *E-* and *Z-*isomers (for both rotamers) is much smaller, since even the less polar (and thus presumably more hydrophobic) *E-*state already exhibits a non-negligible dipole moment. Both calculated rotamer pairs show a similar stability, with rotamer *E*-2 seemingly being slightly more stable than *E*-1, and *Z*-1 seemingly being slightly more stable than *Z*-2. The electronic energies of all rotamers are summarized in the Supporting Information (cf. Table S1). Also, the calculation of the complete AAPEAm molecule gave similar results as for the truncated model shown in Figure 1, i.e., only a small gain in dipole moment is expected upon *E*-to-*Z*-isomerization (cf. Supporting Information Figure S4). Still, the smaller change in dipole moment overlays with a more pronounced *E-* to *Z*-isomerization after UV-light irradiation, which promotes the overall increase in the dipole moment. Note that these calculations did not take into account the surrounding solvent, temperature, or further interactions, e.g., with other chromophores.

We have previously reported the UV-light-induced photoisomerization of monomer AAPEAm in solution from the *E*-state to the metastable *Z*-state. The UV–vis absorption spectra of the *E-* and *Z-*isomers of AAPEAm displayed distinct absorption maxima, and the thermal relaxation of *Z*- to the thermodynamically preferred *E-*state was relatively slow. The half-life of the metastable *Z*-state turned out to be 29 h at 20 °C in methanol.^57^ Now, we demonstrate the reversible and quantitative switching between both isomer states using light of specific wavelengths. The stability of the photoswitch over multiple irradiation cycles is also tested.

As shown in Figure 2, the *E-* and *Z-*states of the azo dye AAPEAm feature distinctive absorption spectra with absorption maxima at 354 nm (π-π* transition) and 440 nm (n-π* transition) for the *E-*isomer, and at 315 and 447 nm, respectively, for the *Z-*isomer. The thermal relaxation after irradiation with UV-light (Figure 2a) takes over 57 h to reach the initial configuration of the sample (dark). This unusually slow thermal relaxation enables quasi-bistable switching of the chromophore in a practical time interval. The sufficiently separated absorption maxima of the *E-* and *Z*-isomers allow for highly selective excitation of each isomer. This enables nearly quantitative switching between the two states by a careful choice of the irradiation wavelengths (Figure 2b). The irradiation with dark blue light (λ = 450 nm) restores only around 70% of the starting configuration (annealed for 1 day in the dark, containing about 2% of *Z*-isomer), although the wavelength is closest to the absorption maximum of the n-π* transition that is more dominant in the *Z*-state. Meanwhile, the irradiation with pale blue light (λ = 480 nm) almost completely restores the initial configuration. The content of the *E-*isomer after the irradiation with green light (λ = 525 nm) even exceeds that of the initial configuration, since the efficient photoisomerization reduces the remaining *Z*-content to <2%.

**Figure 2:**
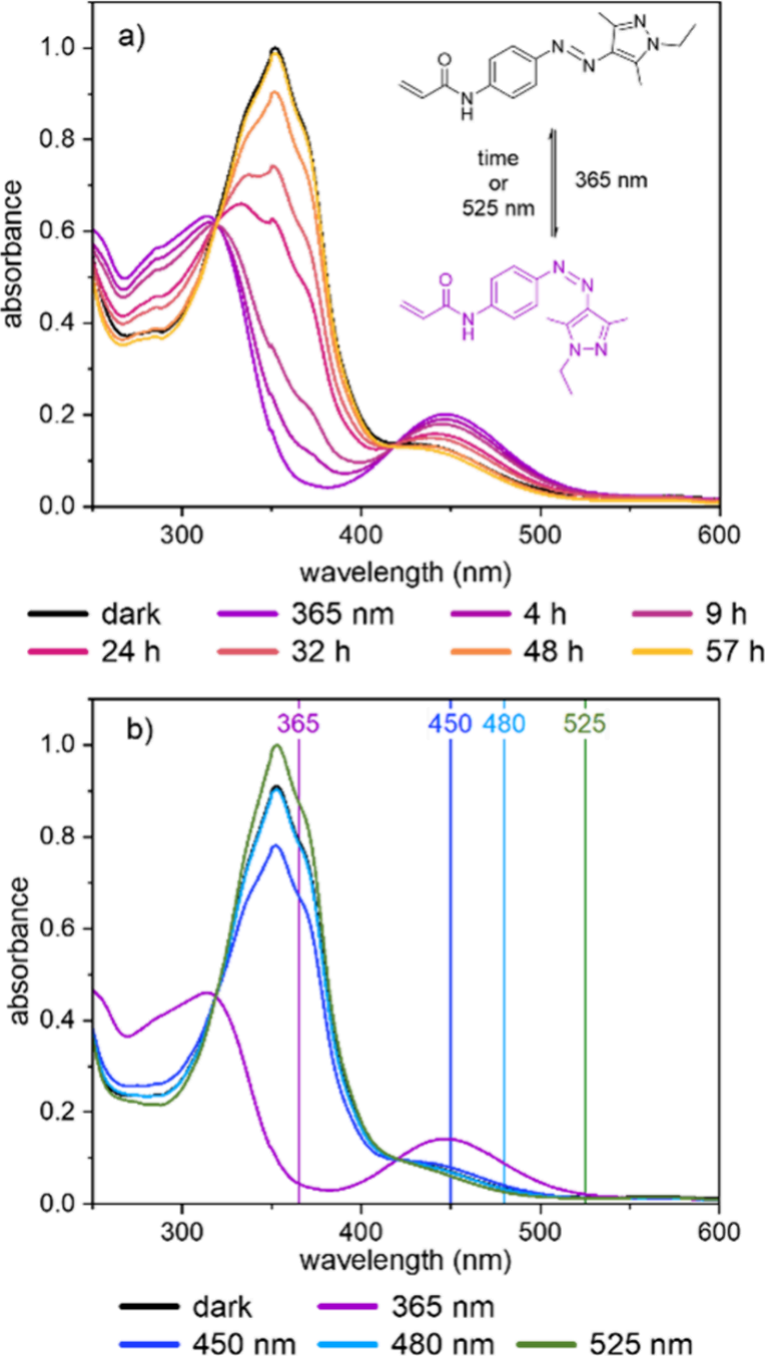
UV–vis spectra of AAPEAm in methanol (1 cm quartz cuvettes, concentration = 0.005 g·L^–1^). The spectra labeled as ″dark″ refer to the samples after annealing for 1 day in the dark before any irradiation is performed. (a) Thermal relaxation of the *Z*-isomer to the *E-*isomer over time after 15 min of irradiation with 365 nm UV-light. The relaxation occurs at ambient temperature in the dark (about 21 °C). The small spike at 350 nm is due to the switch in the irradiation source. (b) Light-induced isomerization of the *Z*-isomer obtained after 15 min of irradiation by 365 nm light (purple) back to the *E-*isomer after irradiation by 450 (dark blue), 480 (pale blue), or 525 nm (green) light. Note that the pale blue and black spectra overlapped nearly perfectly. The colored lines indicate the wavelength at which the sample was irradiated and the maximal absorbance is set to 1.

The results in Figure 2b are obtained under optimal irradiation conditions, i.e., at a low concentration of the chromophore (0.005 g·L^–1^). This ensures that the sample is still partially transparent, which is mandatory for UV–vis spectroscopy experiments. To explore a setting that is more relevant to applications, we repeated the irradiation experiment and recorded a series of ^1^H NMR spectra. An AAPEAm sample solution with a much higher concentration (17 g·L^–1^) is irradiated directly in the NMR tube. While the nonirradiated samples show that mainly the *E-*isomer is present (98%), the photostationary state of the UV-light irradiated sample shows an *E-*state content of <3%, as shown in Table 1. Upon irradiation with 450 and 480 nm light, which induces the back isomerization, we obtain *E-*state contents of 71% and 82%, respectively. Irradiation with green light (525 nm) yields an almost quantitative switching back with an *E*-content of 94%. Although the photoswitching is less effective compared to the UV–vis experiment (cf. Figure 2b), the same general trends are observed, and the switching back to the *E*-state with green light still is nearly quantitative. Table 1 summarizes the *E*-contents of the corresponding irradiation wavelengths.

**Table 1:** *E-*Isomer Content of AAPEAm before and after Irradiation with Different Wavelengths

sample state	*E* content^a^ (%)
1 day annealed at room temperature in the dark	98
365 nm irradiation	3
450 nm irradiation	71
480 nm irradiation	82
525 nm irradiation	94

a Via ^1^H NMR (25 °C, 400 MHz, CD_3_OD).

A number of AAP photoswitches have been known for their quantitative switching behavior with UV- and green light.^49,51,52^ While the substitution pattern on the AAP core can influence the absorption spectra and half-life of the *Z-*isomer, our UV–vis and NMR experiments corroborate that this quality is preserved for the acrylamide derivative AAPEAm. Further, they show that the setting of the irradiation experiments can also influence the effectiveness. It is noteworthy that the most quantitative switching back is not achieved at a wavelength close to the n-π* absorption maximum of the *Z*-state chromophore at about 440 nm, yielding in fact the lowest switch back efficiency of the various wavelengths employed. This finding underlines that instead of the absolute absorption of the *Z-*state chromophore, it is the ratio between the absorption of both isomers at the given wavelength that determines the effectiveness of the photoswitch.

The longevity of the chromophore over several photocycles is illustrated in Figure 3. Starting from a nonirradiated sample, the chromophore dissolved in methanol (17 g·L^–1^) is alternately irradiated with 365 nm UV-light and 525 nm green light using a high-intensity LED system.

**Figure 3:**
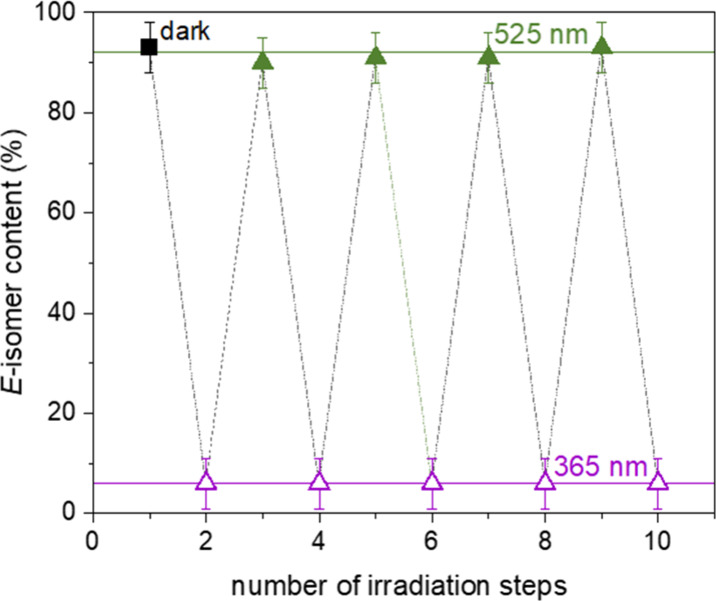
*E-*isomer content of AAPEAm determined by ^1^H NMR (25 °C, 400 MHz, CD_3_OD, concentration = 17 g·L^–1^). The black square shows the *E-*isomer content before irradiation; the colored triangles indicate the content after alternating irradiation by light of either 365 (purple triangle up open) or 525 nm (green triangle up solid). The dotted line is meant as a guide for the eye.

Irradiation at 365 nm always yields an *E*-isomer content of around 5%, while irradiation at 525 nm always yields an *E*-isomer content of over 90%, independent of the irradiation step. The slightly varying *E*-isomer contents obtained after irradiation at 525 nm are attributed to the particular geometry and intensity of the homemade LED setup, limiting the exact reproduction of the irradiation distance and angle. The lack of fatigue is in agreement with prior reports on azopyrazole chromophores^45^ and indicates that the photoisomerization of AAPEAm proceeds without side reactions or similar processes, which inactivate or irreversibly alter the dye. The lack of fatigue is corroborated by turbidity measurements of the corresponding copolymer p­(DMAm-*stat*-AAPEAm) in water, demonstrating the preservation of the thermal transition behavior over 12 irradiation steps within the precision of the measurements (cf. Supporting Information Figure S5).

Following this characterization of the low molecular photoswitch monomer, we investigated the translation of the photoisomerization onto the amphiphilic polymer and its thermo- and light-responsive properties.

### Macroscopic Turbidity Behavior

In our previous work, we briefly described turbidity experiments of aqueous solutions of p­(DMAm-*stat*-AAPEAm) copolymers to study the LCST-type phase transition.^57^ While the absolute *T*_CP_-values of the copolymers decreased with increasing arylazopyrazole content, reflecting the hydrophobicity of the chromophore,^62^ we observed the greatest increase of *T*_CP_ upon irradiation for azo dye contents of 7–9 mol % in the polymer. Lower and higher amounts reduced the extent of the photoinduced temperature shift, Δ*T*_CP_. This finding suggests that at least two opposing effects influence *T*_CP_, with an optimum at 7–9 mol % of the dye. Figure 4 shows the temperature-dependent light transmission of p­(DMAm-*stat*-AAPEAm) solutions (concentration = 10 g·L^–1^, in water) before and after irradiation with 365 nm UV- and 525 nm green light, respectively, as well as the chemical structure of the copolymer in both photoinduced isomeric states. Virtually the same photoinduced *T*_CP_ shift is observed in deuterated water, although *T*_CP_ is reduced for both isomers by around 5 °C (*cf*. Figure S6, Supporting Information). While H-D isotope effects on the phase transition temperature in aqueous solution are well-known, we note that the effect for p­(DMAm-*stat*-AAPEAm) is not only larger but also inverted compared to “gold standard” poly­(*N*-*iso*-propylacrylamide) p­(NiPAm).^63,64^

**Figure 4:**
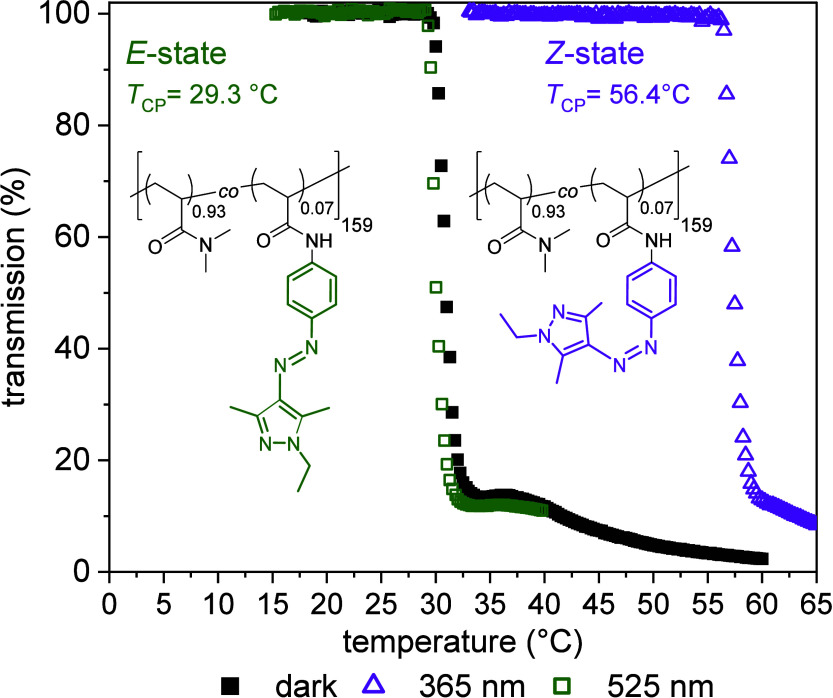
Turbidity curves of p­(DMAm-*stat*-APPEAm) in water (concentration 10 g·L^–1^) before irradiation (annealed 1 day in the dark at ambient temperature, *E-*state, black square solid), after irradiation with 365 nm UV-light (*Z-*state, purple triangle up open), and after isomerization back to the *E-*state by irradiation with 525 nm green light (green square open).

The copolymer p­(DMAm-*stat*-AAPEAm) with 7 mol % of incorporated dye exhibited the strongest change, with a *T*_CP_ shift of Δ*T*_CP_> 25 °C after UV-light irradiation. Such a large shift is, to the best of our knowledge, unprecedented for well-defined, purely photomodulated polymers functionalized with azo dyes, and can be completely reversed by irradiation with 525 nm green light. While these results already make the copolymer highly attractive for diverse applications from photocontrolled molecular valves and sensors to building blocks for controlled drug release systems, a deeper understanding of the system is desirable. The observed *T*_CP_ only represents the temperature at which light of a wavelength of 600 nm (the wavelength used for the turbidimetry) is scattered by large-scale polymer chain aggregation. Thus, *T*_CP_ reports on the aggregation of the polymer only at the macroscopic level and at the start of the phase separation process. The photomodulation of the polymer solubility under isothermal conditions in the temperature window of interest, i.e., between 30 and 56 °C (cf. Figure 4), is exemplified in Figure 5 for the temperature of about 35.5 °C. The changes between clear and turbid solutions (below and above the phase transition, respectively), and yellow and orange colors (the chromophore being in the *E*- or *Z*-state, respectively) are easily visible to the naked eye, e.g., the sample in the *E*-state at 35 °C (cf. Figure 5, picture 2) is yellow and turbid. Additional images visualizing the thermal and photoswitching in deuterated water are shown in the Supporting Information (cf. Figure S7).

**Figure 5:**
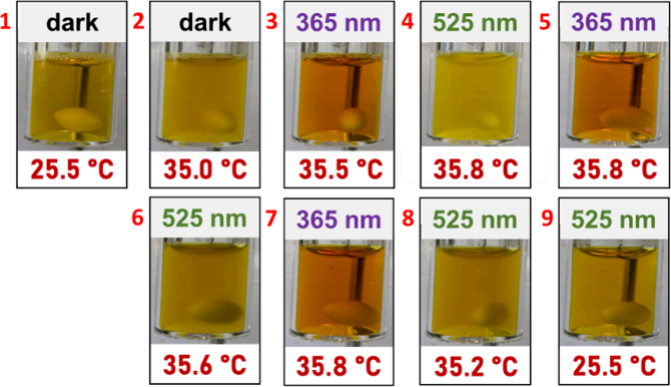
Visualization of the reversible solubility modulation of a solution of p­(DMAm-*stat*-AAPEAm) in water (10 g·L^1^) going from left to right and top to bottom. Pictures 1 and 9 show the polymer dissolved at about room temperature in its less hydrophilic *E*-state. Pictures 2–8 show light-induced solubility switching by alternating irradiation with 365 nm UV and 525 nm green light at about 35.5 °C (*T*_CP_ (*E*-state) < *T* < *T*_CP_ (*Z*-state)).

To gain further insight into the interaction of the polymer or chromophore with its environment and the molecular solvation state of the polymer chain, additional techniques were applied, particularly temperature-resolved electron paramagnetic resonance (EPR) and nuclear magnetic resonance (NMR) spectroscopy.

### Electron Paramagnetic Resonance (EPR) Spectroscopy

EPR spectroscopic experiments describe the chemical or electronic environment of unpaired electron spins, i.e., usually short-lived or persistent radicals, or paramagnetic transition metals. Recently developed EPR methods employ radical functionalized probes, e.g., bearing the 4,4-dimethyloxazolidine-*N*-oxyl (″DOXYL″) moiety, to observe their interaction in solution with macromolecules of interest.^65–67^ Using the amphiphilic probe 5-DOXYL stearic acid, 5-DSA, these methods were applied to obtain more insights into the temperature-dependent solution properties of the copolymer p­(DMAm-*stat*-AAPEAm) when the azo dye is either in the *E*-isomer or *Z*-isomeric state. Hence, for the solution of p­(DMAm-*stat*-AAPEAm) (10 g·L^1^) containing the probe 5-DSA, a series of EPR spectra was recorded in the temperature range of 10–75 °C, increasing the temperature stepwise by 5 °C, and analyzed. The samples were either annealed and measured in the dark, i.e., the AAP moiety was in the *E*-state (Figure 6), or irradiated with 365 nm UV-light, i.e., the AAP moiety was in the *Z*-state (*cf*. Supporting Information, Figure S8). In this way, the respective binding affinities and the effect of the LCST transition were investigated and compared. In the case of the nonirradiated polymer (*E*-state), the spectra at lower temperatures are bimodal with broadening at all spectral positions. These broad features are the hallmark of 5-DSA that is hindered in its rotational motion, as is often observed for amphiphilic spin probes in (bio)­macromolecular solutions or gels.^65–67^ For the UV-light-irradiated solutions at lower temperatures, it is also apparent that the contribution of the broadened 5-DSA spectral features is reduced compared with the same polymer before irradiation.

**Figure 6:**
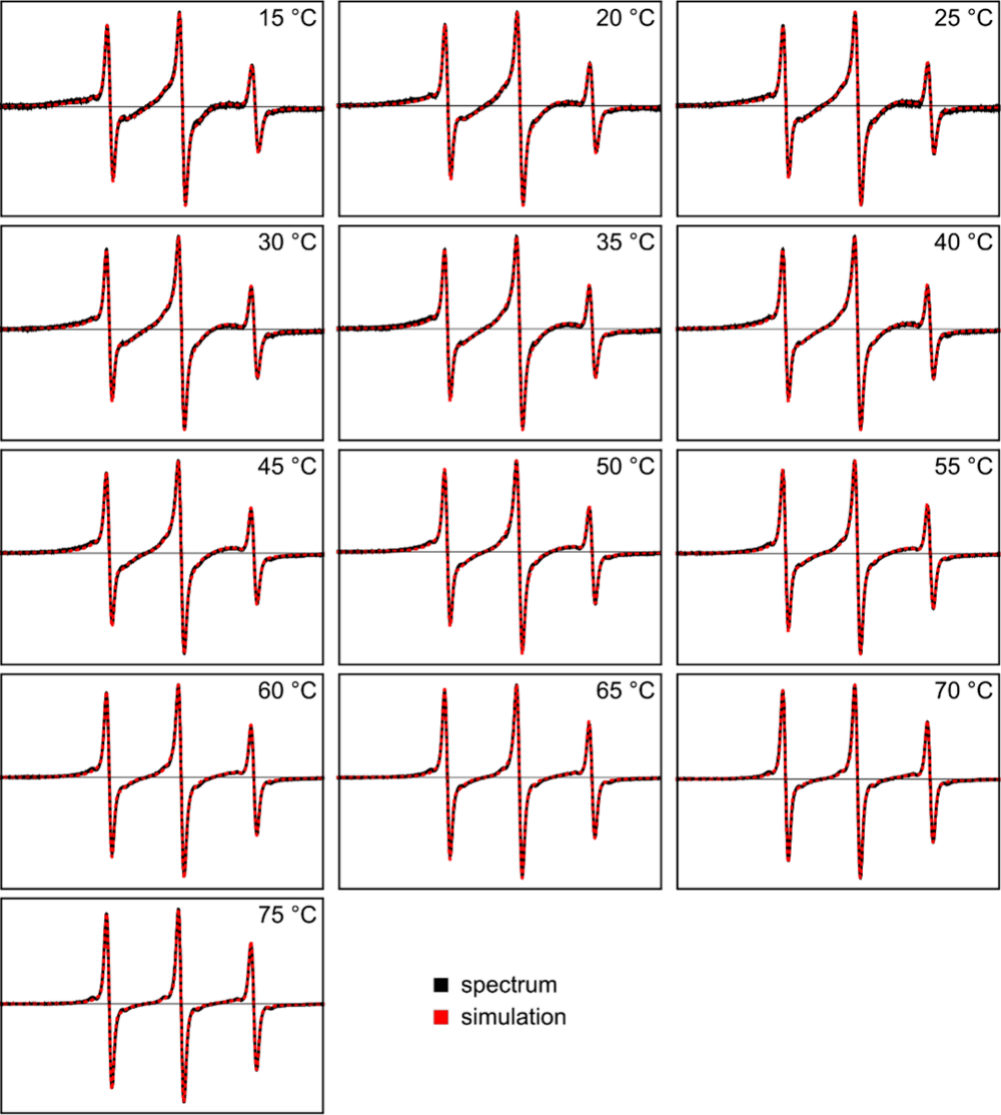
Temperature-dependent EPR spectra and the spectral simulations (black and broken red lines superposed, respectively) of aqueous solutions of p­(DMAm-*stat*-AAPEAm) (10 g·L^1^) when the chromophore is in the *E*-state (sample annealed and kept in the dark). Solutions contain 100 μM spin probe 5-DSA.

Each simulated spectrum results from a combination of two spin systems: a fast-rotating nitroxide radical with rotation correlation times τ_*c*_ of ≈ 0.05–0.2 ns, and a slower rotating nitroxide radical with rotation correlation times τ_*c*_ of ≈1.5–3 ns. The simulation parameters are listed in Tables S2 and S3 in the Supporting Information. The fraction of bound (i.e., interacting) 5-DSA, *X*(5-DSAbound), can be deduced from the simulations. The results are plotted against the temperature in Figure 7 for both the nonirradiated (dark) temperature series and the UV-light-exposed temperature series of the copolymer p­(DMAm-*stat*-AAPEAm), also summarizing the behavior of the probe in the presence of the monomer AAPEAm for comparison. While revealing a maximum of 5-DSA binding (80–90%) to the copolymer at temperatures between 15 and 45 °C, the plot gives no indication of a change in the 5-DSA binding by the polymer when going through the LCST transition. This is true for both EPR spectra series, irrespective of the isomeric state of the azo dye. Yet, for the polymer in the *Z*-state after UV-light irradiation, the maximum fraction of bound 5-DSA is lower by about 1/3 (i.e., at ∼50–60%, cf. Figure 7). For both series, the binding capacities seem to slightly decrease for temperatures above 45 °C. This may be due to the elevated kinetic energies of the dissolved 5-DSA probe and polymer molecules.

**Figure 7:**
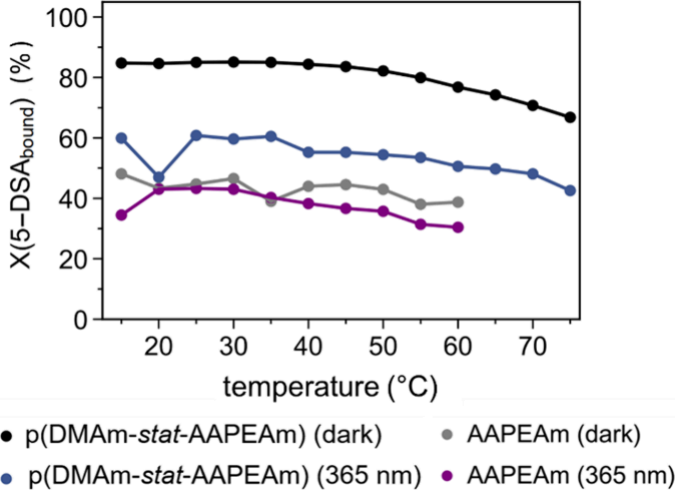
Effect of the isomeric state of the azo dye on the temperature-dependent percentage of the probe 5-DSA (*X*(5-DSA_bound_)) bound by the copolymer p­(DMAm-*stat*-AAPEAm): upper curves, black circles = *E*-state (sample annealed in the dark), blue circles = *Z*-state (sample irradiated with 365 nm UV-light). For comparison, analogous measurements for the monomer AAPEAm are included: lower curves, gray circles = *E*-state (sample annealed in the dark), and purple circles = *Z*-state (sample irradiated with 365 nm UV-light). The lines are intended as a guide to the eye. Data was obtained from spectral simulations as displayed in Figure 6 (see also Figure S8, Supporting Information).

The findings are remarkable from the EPR spectroscopic and polymer perspective. Using the amphiphilic stearic acid derivative 5-DSA, differences in the binding capacity to the polymer p­(DMAm-*stat*-AAPEAm) between the azo dye in the *Z*-state with a high dipole moment and being in the *E-*state with a low dipole moment are clearly observable. The reduced binding affinity to the amphiphilic probe when the azo dye is in the *Z*-state coincides with the *Z*-state’s higher polarity and, thus, its lower hydrophobicity compared to the *E*-state. Furthermore, the binding due to the rather low content of azo dye, namely of only 7%, being either in the higher or in the lower polarity states, apparently outweighs the effect of the copolymer’s thermal response, as we often observed using this EPR spectroscopic approach.^65–67^ This finding may have important consequences, e.g., for designing such stimuli-sensitive polymers for their use in controlled drug delivery.

### Liquid-State Nuclear Magnetic Resonance (NMR) Spectroscopy

The molecular dynamics and solvation of the polymer p­(DMAm-*stat*-AAPEAm) in aqueous solution is followed by a series of ^1^H NMR spectra for each of the photoisomeric states. Spectra were recorded within the window of 20–80 °C for the temperature rising in steps of 5 °C using DSS-D_6_ as the internal standard. The results of both series for the range between 3.5 and 0.5 ppm are shown in Figure 8 (for a full spectrum, see ref. 57), and compared for the evolution of the relative signal intensity with temperature to the results obtained from turbidimetry. An example of the turbidimetry data of p­(DMAm-*stat*-AAPEAm) in deuterated water is shown in the Supporting Information (*cf*. Figure S6). The analogous analysis of the temperature-dependent intensity of the aryl proton signals in the range between 8.2 and 6.8 ppm reveals the identical behavior, as shown in the Supporting Information (cf. Figure S9).

**Figure 8:**
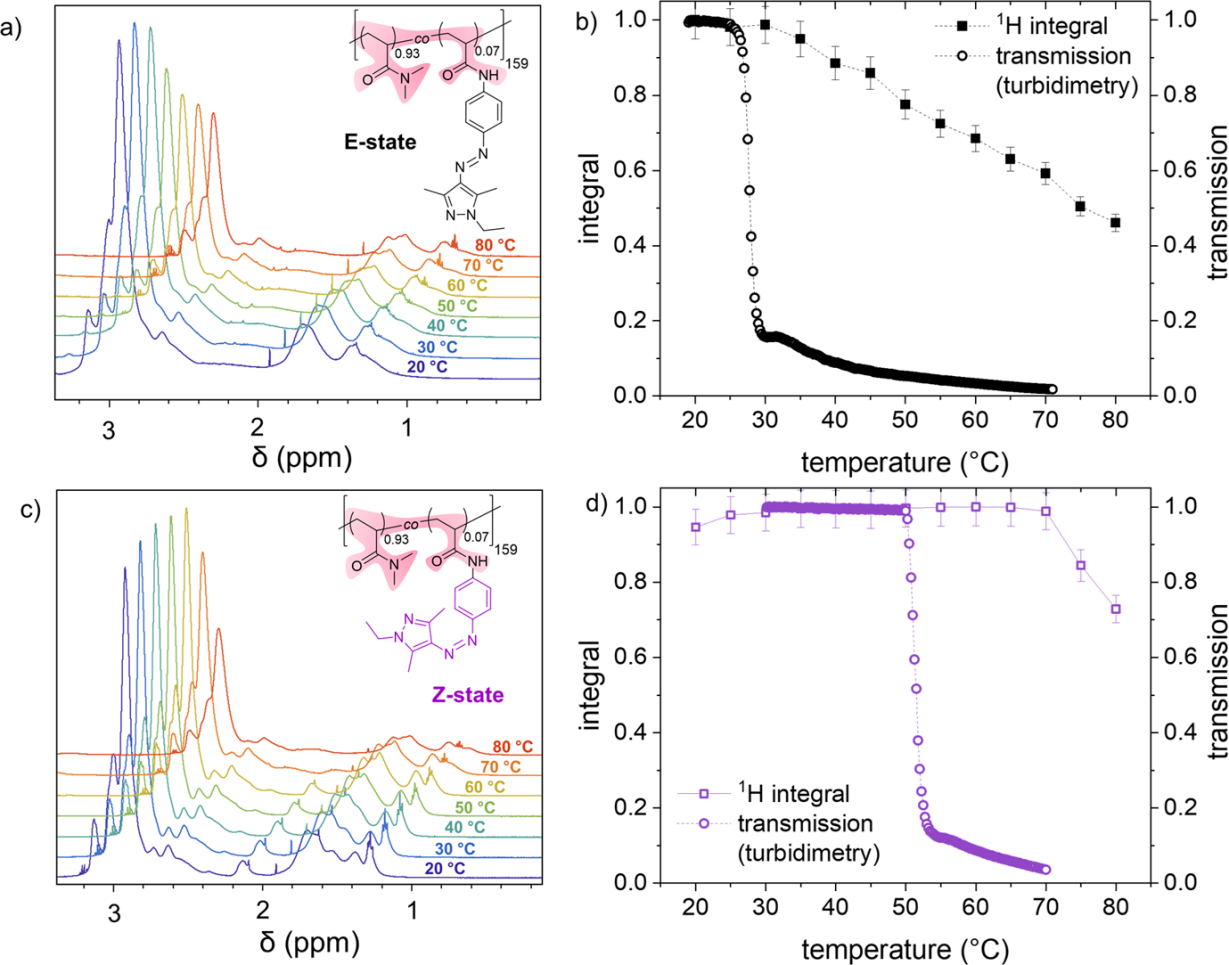
Temperature-dependent ^1^H NMR spectra of the copolymer p­(DMAm-*stat*-AAPEAm) in D_2_O (concentration 10 g·L^1^): (a) the nonirradiated (*E-*state) and (c) the UV-light irradiated sample (*Z*-state), shown in 10 °C steps. Normalized decay of the integral of the NMR signals from the polymer backbone and the dimethyl amino group (highlighted by pink color in the formulae) as a function of temperature (squares) compared to the temperature-dependent turbidity (circles) (b) in the *E-*state and (d) in the *Z*-state sample.

Characteristically, the cumulative integral of the polymer NMR signals remains constant up to a specific temperature, *T*_decay_, above which the intensity decays continuously. This decay of the polymer NMR signals can be interpreted as the consequence of the progressive removal of the well-hydrated and therefore freely moving and rotating polymer chains or their segments from the aqueous homogeneous phase and the simultaneous increasing amount of polymer in the polymer-rich microphase. In the latter, the polymer chains or their segments become increasingly dehydrated as the temperature rises, thus reducing their mobility. Accordingly, NMR spectroscopy reports on the solvation behavior of the polymer chains at the molecular level. This complements the information on the macroscopic behavior from turbidimetry, which is primarily sensitive to the onset of the phase separation, as indicated by the cloud point.

We note that the onset of the signal decay at *T*_decay_ is shifted compared to the measured *T*_CP_ values in D_2_O, namely 30 °C for the *E-*state sample and 70 °C for the *Z*-state sample, versus 25 and 50 °C, respectively, for the corresponding *T*_CP_ values in D_2_O. The 5 °C step resolution of the temperature-resolved NMR spectra is lower than the 0.25 °C step resolution of the turbidimetry. Still, the differences are too large to be attributed solely to this factor. While both samples display a *T*_CP_ lower than the onset of the NMR signal decay, the discrepancy between the observed transition temperatures *T*_CP_ and *T*_decay_ is rather small and may possibly still be within the margin of the measurement inaccuracy for the nonirradiated sample in the *E*-state (Δ­(*T*_decay_ – *T*_CP_) = ∼5 °C). However, the discrepancy is much larger for the irradiated sample in the *Z*-state (Δ­(*T*_decay_ – *T*_CP_) = ∼20 °C).

A possible explanation for the differences between *T*_CP_ and *T*_decay_ could be the nature of the coil-to-globule phase transition of p­(DMAm-*stat*-AAPEAm). In the case of the DMAm-based polymers, it is of the so-called LCST-type I, *i.e*., of the classical Flory–Huggins type. Whereas in the case of the exceptional so-called LCST behavior of type II, which is best documented for poly­(*N*-*iso*-propylacrylamide) (pNiPAm),^68^ the phase transition goes along with a step change of the hydration and a discontinuous shrinkage of the polymer. In the case of LCST behavior of type I, only a continuous, gradual reduction of hydration with a continuous shrinking takes place.^69^ Consequently, and in contrast to p­(NiPAm),^70,71^ the degree of dehydration that translates into the sufficiently reduced polymer mobility required to induce a noticeable decay of the NMR signal intensity can only be achieved at temperatures higher than *T*_CP_. It also seems consistent with this line of reasoning that the polymer containing the more polar hydrophobe, namely, the *Z*-isomer of AAPEAm, undergoes less efficient dehydration with increasing temperature, thus increasing the difference between *T*_CP_ and *T*_decay_.

## Conclusions

The statistical copolymer p­(DMAm-*stat*-AAPEAm) prepared from the nonionic monomer *N,N*-dimethyl acrylamide DMAm and the functional acrylamide AAPEAm bearing an aryl azopyrazole dye is investigated concerning its combined thermo- and photoresponsive behavior in aqueous solution. The dual responsive copolymer system, which undergoes an LCST-type coil-to-globule transition as well as an effective *E*-to-*Z* photoisomerization, is shown to be a promising alternative to the previously used thermo- and photoresponsive polymers bearing azobenzene dyes. Compared to the latter, the new system excels by the relatively long-lived metastable *Z* state (half-life > 1 d) and by the nearly quantitative conversion of the *E*- into the *Z*-state and *vice versa* upon irradiation with a near-UV-light of 365 nm or green light of 525 nm, respectively.

Remarkably, the cloud point of its 1 wt % aqueous solution is shifted from about 29 to 56 °C upon *E*-to-*Z* photoisomerization, which, to our knowledge, represents an unprecedented high cloud point change for azo dye-functionalized photoswitchable polymers. The reasons for this exceptionally large effect are not clear. According to the common understanding of the photoswitching of azo dye-functionalized polymers, the increase in the phase transition temperature is caused by the rise of the azo dye’s polarity in the *Z*-state. However, computer modeling shows that the difference in the dipole moment between the *E*- and the *Z* -states is smaller for the new arylazopyrazole dyes than for the classical azobenzene-derived dyes. Notwithstanding the yet missing explanation for the phenomenon, the -compared to previous azodye-functionalized polymers- extremely broad temperature window between the cloud points of the polymer in the *E*- and Z-states represents a major progress, and opens up many opportunities for isothermal photoswitching in practical contexts.

Crucially, the photoswitching of the cloud point shows no fatigue over multiple irradiation cycles. Also, the resulting large temperature window enables an effective isothermal photoswitching, e.g.*,* at human body temperature. Temperature-dependent EPR studies with the amphiphilic probe 5-DOXYL stearic acid (5-DSA) showed that the *E*-to-*Z* photoisomerization, even at a low-to-moderate azo dye content of 7 mol %, affects the interaction with the probe more than the coil-to-globule phase transition. Temperature-dependent ^1^H NMR studies also suggest that the dehydration of the copolymer above the coil-to-globule phase transition occurs rather gradually and that the mobility of the polymer segments is significantly reduced only at temperatures well above the cloud point. This is particularly true for the polymer in the more polar *Z*-state. These findings should be taken into account in the design of stimuli-responsive polymers derived from p­(DMAm-*stat*-AAPEAm) copolymers, e.g., for the controlled delivery of active agents or drugs.

## Supplementary Material



## Notes

^1^H NMR spectra of the *E*- and *Z*-isomers of the dye monomer; details on the modeling of the dipole moments; more detailed characterization of the photoisomerization and the resulting modulation of the phase transition behavior by visual inspection, turbidimetry, and ^1^H NMR; details on the EPR spectra and spectrum simulations (PDF)
