# Supplementary material for: Toward Fully Photoresponsive Amphiphilic Polymers via Azopyrazole-Functionalized Polyacrylamides
Source: Macromolecules. 2025 Oct 8;58(20):11088–98. doi: 10.1021/acs.macromol.5c01176 (PMC12573787; doi:10.1021/acs.macromol.5c01176)
Supplement: Supplementary file 1 [file ma5c01176_si_001.pdf]

Supporting Information

Toward Fully Photoresponsive Amphiphilic Polymers via Azopyrazole-Functionalized Polyacrylamides

René Steinbrecher [a], Florian Lehmann [b], Ruslan Nedielkov [a], Tillmann Klamroth [a],

Heiko Möller [a], Andreas Taubert [a], Dariush Hinderberger [b], Peter Müller-Buschbaum [c],

*Christine M. Papadakis [c], and André Laschewsky [a,d]**

AUTHOR ADDRESS:

[a] Institute of Chemistry, University of Potsdam, 14476 Potsdam-Golm, Germany.
E-mail: laschews@uni-potsdam.de.

[b] Institute of Chemistry, Martin Luther University Halle-Wittenberg, 06099 Halle (Saale), Germany

[c] Department of Physics, TUM School of Natural Sciences, Technical University of Munich, 85748 Garching, Germany.

[d] Fraunhofer Institute for Applied Polymer Research IAP, 14476 Potsdam-Golm, Germany

^{1}H NMR spectra for the determination of the ratio between *E*- and *Z*-isomer

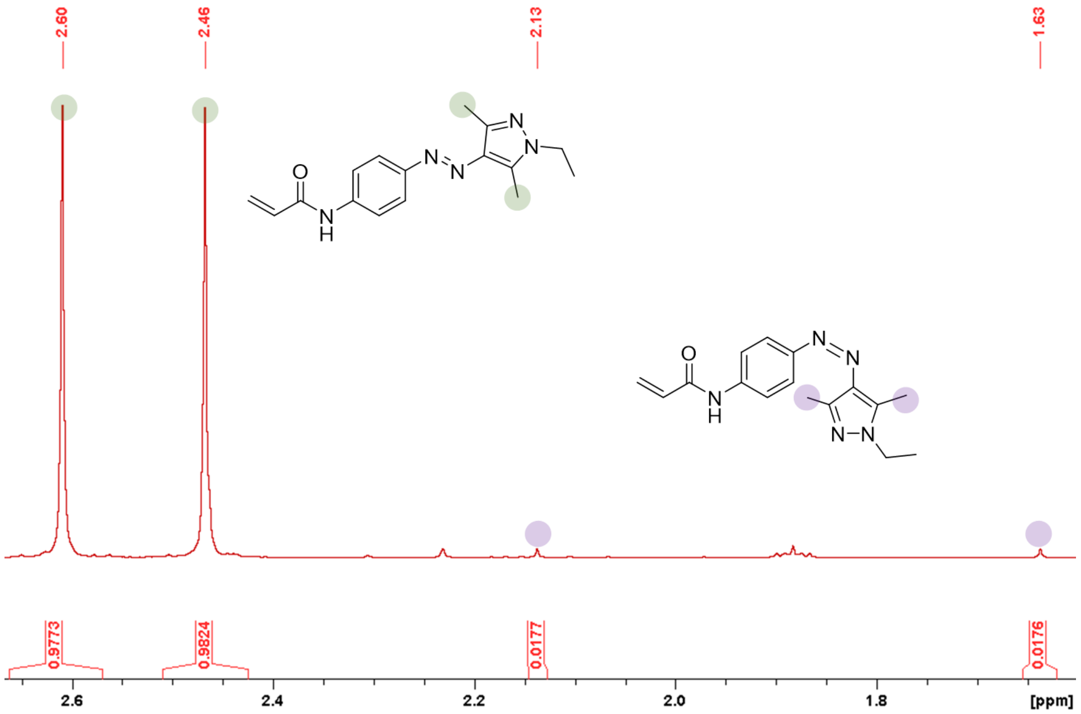

Figure S1. ^{1}H NMR spectrum (400 MHz) of the monomer AAPEAm in deuterated methanol at room temperature before irradiation at 365 nm. The integrals of the corresponding *E* and *Z* states are normalized to 1 to calculate the ratio of *E : Z* (98:2). Colored circles (green and purple) show the corresponding signals.

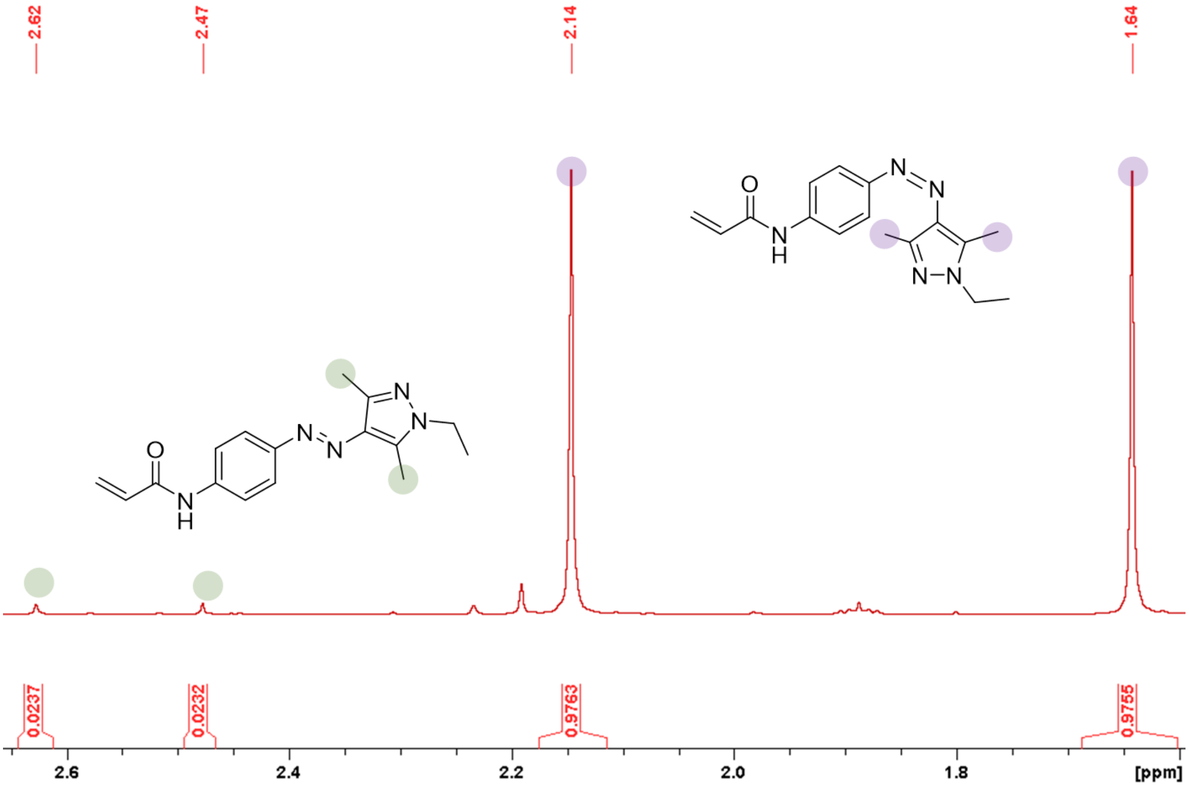

Figure S2. ^{1}H NMR spectrum (400 MHz) of the monomer AAPEAm in deuterated methanol CD_3OD at room temperature after irradiation at 365 nm. The integrals of the corresponding *E* and *Z* states are normalized to 1 to calculate the ratio of *E : Z* (2:98). Colored circles (green and purple) show the corresponding signals.

Supplementary DFT data

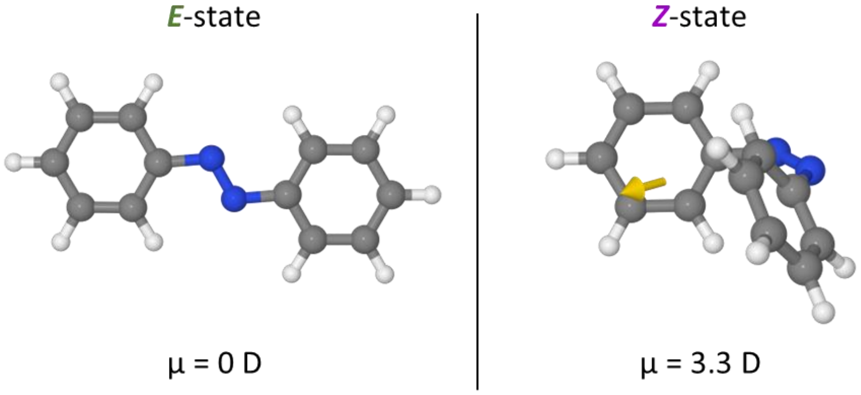

Figure S3. Calculated dipole moments of unsubstituted azobenzene in the *E*-and *Z*-state (calculations at the PWPB95-D3/def2-TZVP level of theory).

Table S1. Electronic energy of the 4 rotamers of Fig. 1 relative to the electronic energy of the most stable rotamer *E*-2 in KJ·mol^{-1}.

Rotamer of truncated AAP model	Relative electronic energy (KJ·mol^{-1})
E-1	1.47
E-2	0
Z-1	46.01
Z-2	46.31

We also performed calculations on the full monomer to verify the findings of the reduced model. We used GOAT (Global Optimization Algorithm) as provided by Orca 6.0.1[1] on the XTB level of theory[2,3] (with FREEZECISTRANS TRUE) to identify possible *Z* and *E* conformers. That yields 37 *E* and 78 *Z* conformers. Each conformer was re-optimized with r^{2}SCAN-3c.[4] For each *Z* and *E*, the ten conformers, which are lowest in energy on the r^{2}SCAN-3c level of theory, were finally optimized and checked by a frequency calculation with the ωB97M-D4[5,6] functional using the def2-TZVP basis set.[7] Here, we use the ωB97M-D4/def2-TZVP level of theory, because this level is especially suitable to determine relative energies[8] and gives results very close to PWPB95-D3/def2-TZVP for the reduced system regarding the dipole moments, with a maximal deviation of 5 %. The *Z* and *E* conformers with lowest electronic energy are shown in Fig. S4. Here, we observe a dipole moment change of about 0.9 D, which agrees well with the findings of the reduced model.

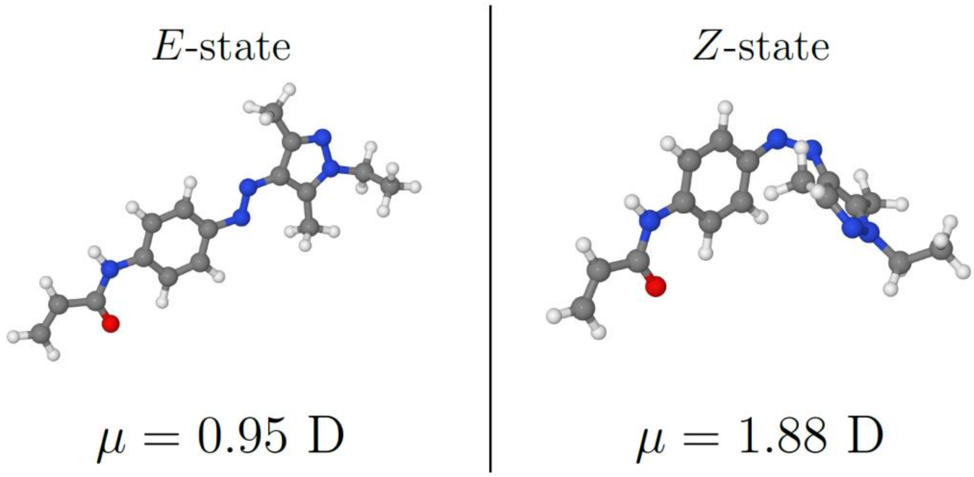

Figure S4. Optimized Z and E conformers of the monomer AAPEAm with lowest electronic energy on the ωB97M-D4/def2-TZVP level of theory.

Supplementary turbidity data

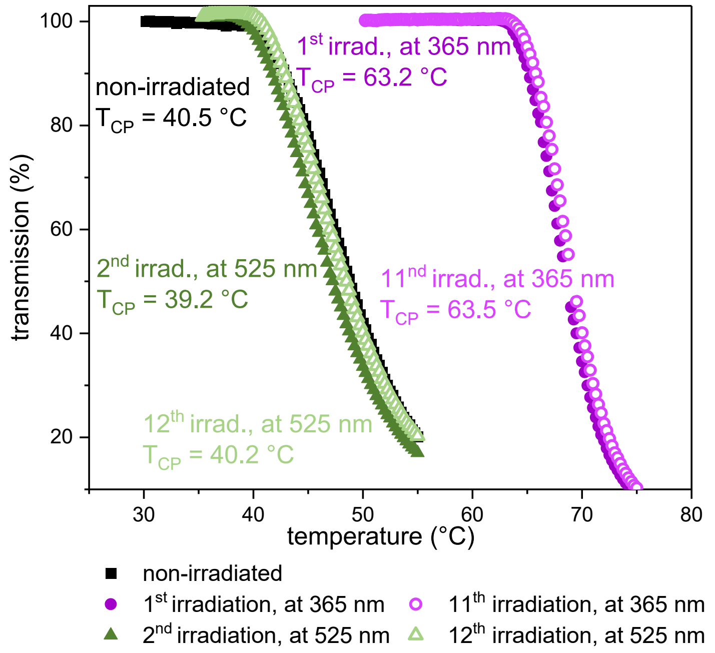

Figure S5. Turbidity measurements of p(DMAm-*stat*-AAPEAm) in H_2O (concentration = 1 g·L¬1). Before irradiation (non-irradiated, black squares), after its first irradiation at 365 nm (UV-light, purple points) and the subsequent irradiation at 525 nm (green light, green triangles). Afterwards, the sample was 8 times alternatingly irradiated with UV and green light, and the turbidity curve of the sample after the 11th (light purple circles) and 12th (light green hollow triangles) irradiation step is shown.

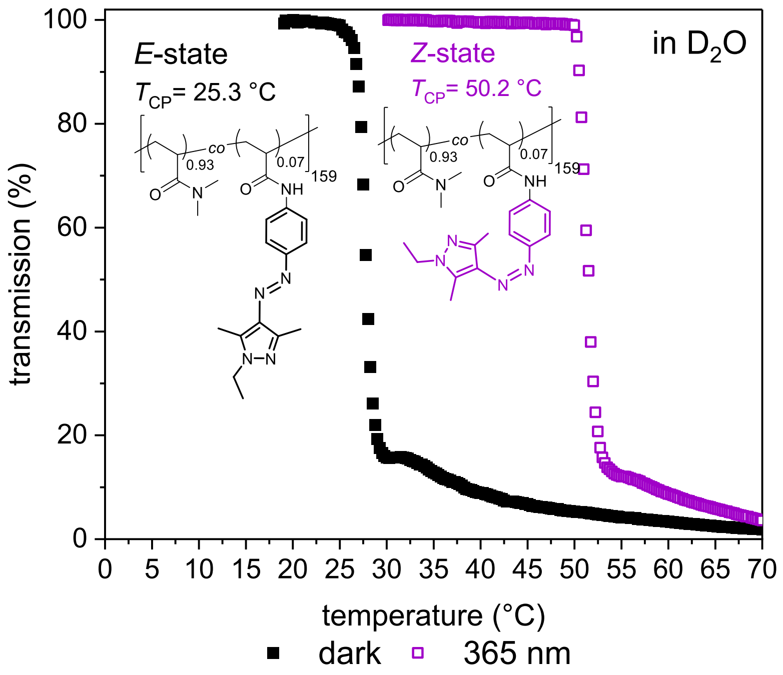

Figure S6. Turbidity measurements of p(DMAm-*stat*-AAPEAm) in deuterated water D_2O (concentration = 10 g·L^{-1}). Before irradiation (dark, black squares) and after irradiation with UV-light (365 nm, purple hollow squares).

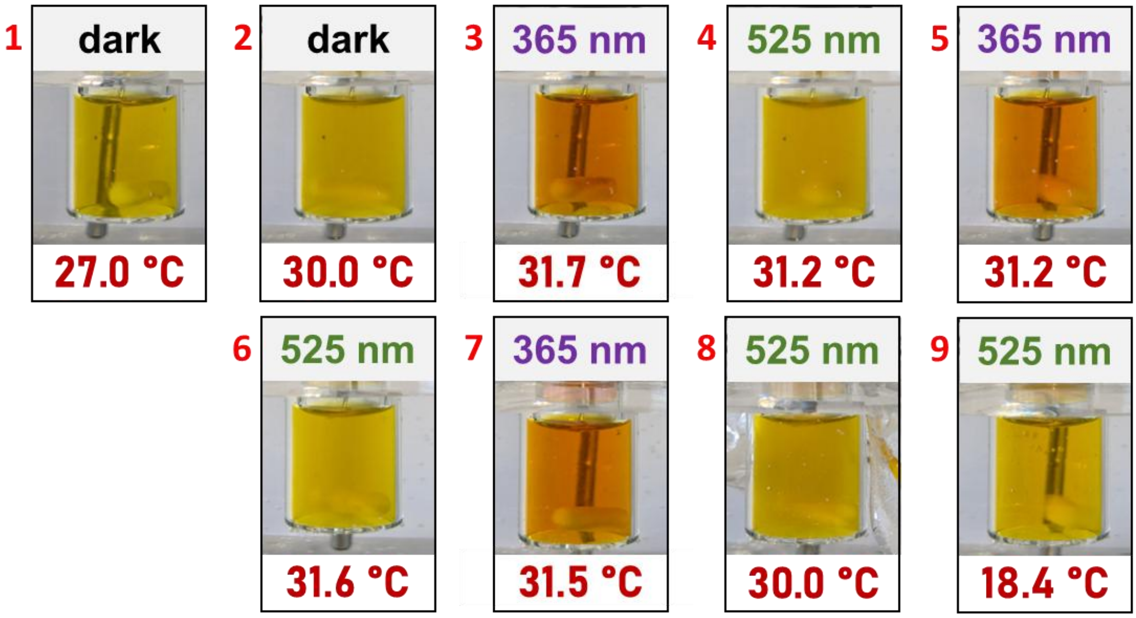

Figure S7. Demonstration of the reversible thermo- and photo-responsive modulation of the solubility of p(DMAm-*stat*-AAPEAm) in D_2O (concentration = 10 g·L^{-1}) with UV (λ = 365 nm) and green (λ = 525 nm) light. Pictures 1 and 2 show thermo-responsive modulation of the solubility of the *E*-state sample before irradiation. Pictures 3-8 illustrate the photo-responsive modulation of the solubility upon alternating irradiation with UV and green light. Picture 9 shows the sample in the *E*-state after the photo cycling and subsequent cooling back to temperatures below the T_{CP}.

Supplementary EPR data

Table S2. *Easyspin* simulation parameters of the simulated systems shown in Fig. 6 in the main text. The ’free’ and ’bound’ simulations at one temperature are combined to yield the final simulated spectrum.

T/°C	component	proportion/%	g_{iso}	A_{iso}	τ_c/ns
15	Free	15.2383	2.00463	44.1484	0.155451
15	Bound	84.7617	2.00463	42.2149	2.95221
20	Free	15.3829	2.00468	44.0829	0.258759
20	Bound	84.6171	2.00464	42.5924	3.09737
25	Free	15.0033	2.00468	44.0723	0.16908
25	Bound	84.9967	2.00466	42.7628	2.93354
30	Free	14.8993	2.00468	44.0571	0.159546
30	Bound	85.1007	2.00469	42.7251	2.81391
35	Free	14.9979	2.0047	44.041	0.214093
35	Bound	85.0021	2.00471	42.9834	2.70162
40	Free	15.6275	2.00471	44.0728	0.174859
40	Bound	84.3725	2.00472	43.0956	2.81536
45	Free	16.4069	2.00469	43.9908	0.163031
45	Bound	83.5931	2.00472	42.6806	2.54962
50	Free	17.8325	2.0047	43.9979	0.141254
50	Bound	82.1675	2.00478	42.6702	2.20222
55	Free	20.0734	2.00469	44.0175	0.0830192
55	Bound	79.9266	2.00474	42.2404	2.32541
60	Free	23.1946	2.0047	44.0081	0.0799121
60	Bound	76.8054	2.00479	42.1632	2.25804
65	Free	25.7565	2.00472	44.0014	0.0938898
65	Bound	74.2435	2.00483	42.4542	1.52894
70	Free	29.2612	2.00472	43.9553	0.123674
70	Bound	70.7388	2.00476	41.7591	1.7629
75	Free	33.2013	2.00473	43.9387	0.105005
75	Bound	66.7987	2.00482	41.7568	1.76632

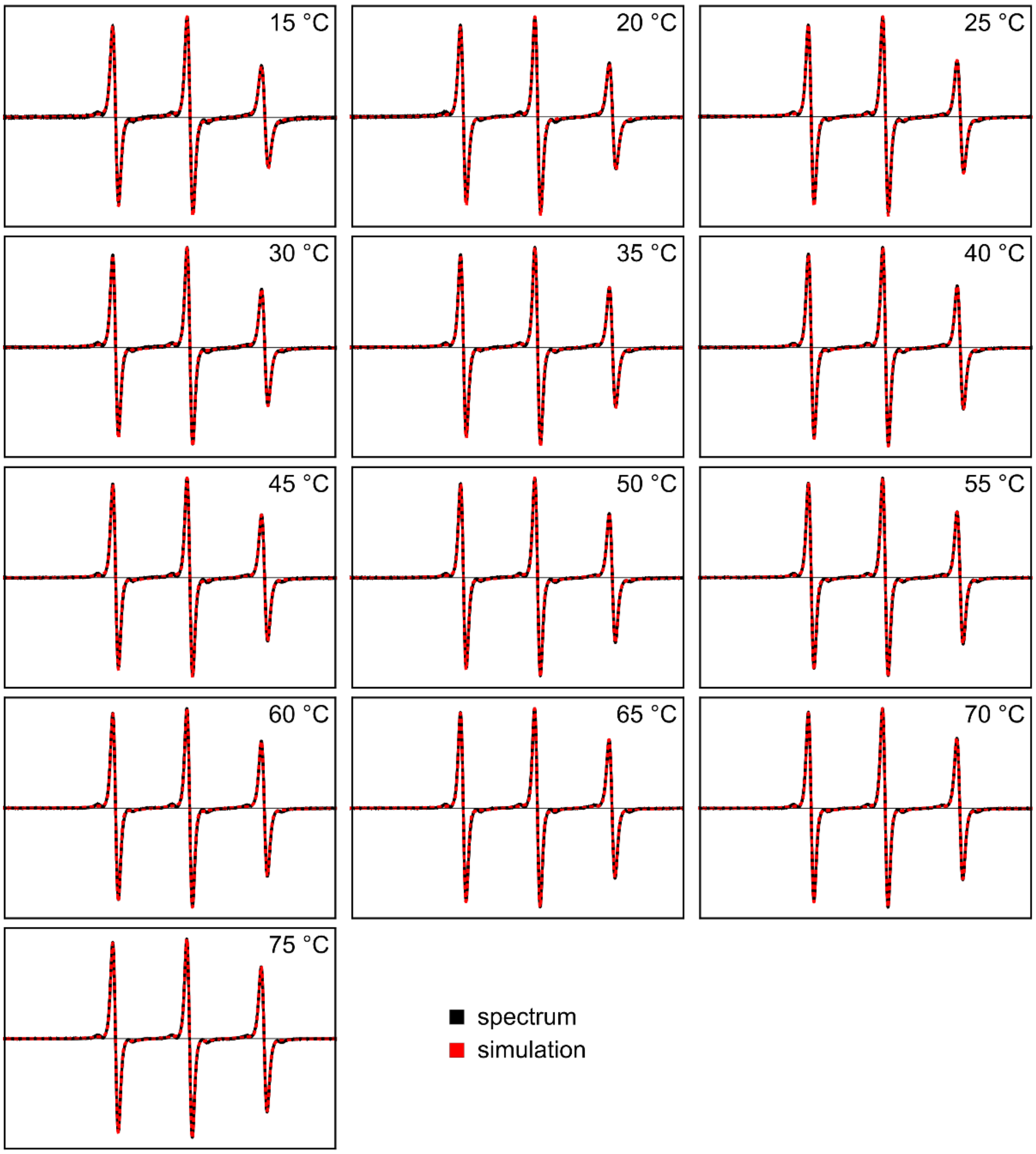

Figure S8. Experimental EPR spectra (black) and the *easyspin* EPR spectrum simulations (broken red line) of a 10 g·L^{-1} polymer p(DMAm-*stat*-AAPEAm) solution in water with 100 µM od probe 5-DSA, recorded during UV irradiation (365 nm) at different temperatures. Each simulated spectrum is a combination of two spin systems: one fast-rotating nitroxide radical and one slower rotating nitroxide radical, which is considered to be polymer-bound. The simulation parameters are listed in Tab. S3.

Table S3. *Easyspin* simulation parameters of the simulated systems shown in Fig. S8. The 'free' and 'bound' simulations at one temperature are combined to yield the final simulated spectrum.

T/°C	component	proportion/%	g_{iso}	A_{iso}	τ_c/ns
15	Free	40.0777	2.00464	44.0693	0.220342
15	Bound	59.9223	2.00449	35.8612	2.7283
20	Free	53.011	2.00465	44.054	0.168784
20	Bound	46.989	2.00449	35.8612	2.7283
25	Free	39.1924	2.00466	44.0988	0.117442
25	Bound	60.8076	2.00478	35.7353	2.70585
30	Free	40.3497	2.00469	44.0651	0.204971
30	Bound	59.6503	2.00459	37.9632	1.89471
35	Free	39.4978	2.00468	44.0589	0.145693
35	Bound	60.5022	2.0046	37.8868	1.71536
40	Free	44.7664	2.00468	44.0279	0.144794
40	Bound	55.2336	2.00454	39.429	1.88878
45	Free	44.7671	2.00468	44.01	0.0924839
45	Bound	55.2329	2.00469	37.8619	2.30539
50	Free	45.5664	2.00468	43.9819	0.095647
50	Bound	54.4336	2.00464	38.8836	1.68167
55	Free	46.4994	2.0047	43.9889	0.101489
55	Bound	53.5006	2.00475	38.8053	1.6803
60	Free	49.4155	2.00469	43.9153	0.0748079
60	Bound	50.5845	2.0047	38.9989	1.72242
65	Free	50.2746	2.00472	43.9125	0.116937
65	Bound	49.7254	2.00467	39.9646	1.23348
70	Free	51.8886	2.00471	43.9276	0.0903553
70	Bound	48.1114	2.00463	39.5545	1.51068
75	Free	57.4298	2.00471	43.95	0.0611608
75	Bound	42.5702	2.0047	40.0677	1.3327

Supplementary Temperature-resolved NMR data

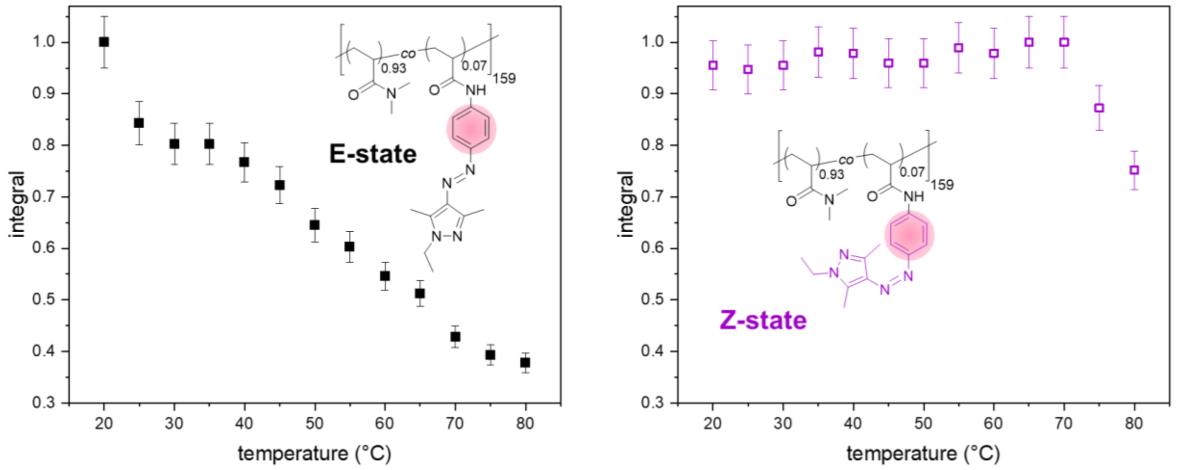

Figure S9. Normalized ^{1}H signal decay of the aromatic ring (marked in pink color) signal of p(DMAm-*stat*-AAPEAm) dissolved in D_2O (concentration = 10 g·L^{-1}) vs. temperature in 5°C steps, using deuterated sodium trimethylsilylpropanesulfonate (DSS-D_6) as internal standard.

References

(1) Neese, F. Software update: The ORCA program system—Version 5.0. *WIREs Comput. Mol. Sci.* **2022**, *12* (5). DOI: 10.1002/wcms.1606.

(2) Bannwarth, C.; Ehlert, S.; Grimme, S. GFN2-xTB-An Accurate and Broadly Parametrized Self-Consistent Tight-Binding Quantum Chemical Method with Multipole Electrostatics and Density-Dependent Dispersion Contributions. *J. Chem. Theory Comput.* **2019**, *15* (3), 1652–1671. DOI: 10.1021/acs.jctc.8b01176.

(3) Bannwarth, C.; Caldeweyher, E.; Ehlert, S.; Hansen, A.; Pracht, P.; Seibert, J.; Spicher, S.; Grimme, S. Extended tight-binding quantum chemistry methods. *WIREs Comput. Mol. Sci.* **2021**, *11* (2). DOI: 10.1002/wcms.1493.

(4) Grimme, S.; Hansen, A.; Ehlert, S.; Mewes, J.-M. r2SCAN-3c: A "Swiss army knife" composite electronic-structure method. *J. Chem. Phys.* **2021**, *154* (6), 64103. DOI: 10.1063/5.0040021.

(5) Caldeweyher, E.; Ehlert, S.; Hansen, A.; Neugebauer, H.; Spicher, S.; Bannwarth, C.; Grimme, S. A generally applicable atomic-charge dependent London dispersion correction. *J. Chem. Phys.* **2019**, *150* (15), 154122. DOI: 10.1063/1.5090222.

(6) Mardirossian, N.; Head-Gordon, M. ωB97M-V: A combinatorially optimized, range-separated hybrid, meta-GGA density functional with VV10 nonlocal correlation. *J. Chem. Phys.* **2016**, *144* (21), 214110. DOI: 10.1063/1.4952647.

(7) Weigend, F.; Ahlrichs, R. Balanced basis sets of split valence, triple zeta valence and quadruple zeta valence quality for H to Rn: Design and assessment of accuracy. *Phys. Chem. Chem. Phys.* **2005**, *7* (18), 3297–3305. DOI: 10.1039/b508541a.

(8) Najibi, A.; Goerigk, L. DFT-D4 counterparts of leading meta-generalized-gradient approximation and hybrid density functionals for energetics and geometries. *J. Comput. Chem.* **2020**, *41* (30), 2562–2572. DOI: 10.1002/jcc.26411.
